# Interleukin-6 promoter polymorphism interacts with pain and life stress influencing depression phenotypes

**DOI:** 10.1007/s00702-016-1506-9

**Published:** 2016-01-28

**Authors:** David Kovacs, Nora Eszlari, Peter Petschner, Dorottya Pap, Szilvia Vas, Peter Kovacs, Xenia Gonda, Gyorgy Bagdy, Gabriella Juhasz

**Affiliations:** Department of Pharmacodynamics, Faculty of Pharmacy, Semmelweis University, Nagyvarad ter 4., Budapest, 1089 Hungary; MTA-SE Neuropsychopharmacology and Neurochemistry Research Group, Hungarian Academy of Sciences, Nagyvarad ter 4., Budapest, Hungary; National Institute of Oncology, Rath Gyorgy u. 7-9, Budapest, Hungary; School of Mental Health Sciences, Semmelweis University, Balassa u. 6, Budapest, Hungary; Department of Clinical and Theoretical Mental Health, Kútvölgyi Clinical Center, Semmelweis University, Kútvölgyi u.4, Budapest, Hungary; Neuroscience and Psychiatry Unit, University Manchester, Manchester, M13 9PT UK; MTA-SE-NAP B Genetic Brain Imaging Migraine Research Group, Hungarian Academy of Sciences, Semmelweis University, Nagyvarad ter 4., Budapest, Hungary

**Keywords:** Interleukin-6, Polymorphism, Pain, Stress, Depression, Zung Self-rating Scale

## Abstract

Interleukin-6 (IL-6) has emerged as a potent biomarker for depression as its elevated plasma levels in patients with clinical depression have been confirmed by meta-analyses. Increased plasma IL-6 concentration was associated with various psychological stress factors and physical disorders accompanied by pain. Another modulator of the IL-6 level is rs1800795, a promoter polymorphism in the *IL*-*6* gene which is able to influence its expression rate. Therefore, we examined in a Hungarian population sample of 1053 volunteers with European origins if rs1800795 polymorphism can affect depression symptoms measured by Zung Self-rating Depression Scale (ZSDS), and Brief Symptom Inventory (BSI). We also investigated the interactions of the polymorphism with reported painful physical conditions and Recent Negative Life Events (RLE) measured by the List of Life Threatening Experiences. Rs1800795 significantly interacted with both RLE and painful condition on depressive symptoms measured by ZSDS and BSI using different heritability models, while no main effects of the polymorphism were identified. After correction for multiple testing only the rs1800795 × RLE interaction effect (recessive model) remained significant on the BSI score, while both RLE and painful conditions significantly interacted on the ZSDS. In conclusion, the functional *IL*-*6* rs1800795 polymorphism in interaction with various stress factors increases the risk of depression and has a greater impact on symptoms measured by the ZSDS. Thus, IL-6 and other cytokines may be more relevant in the development of somatic symptoms compared to affective signs of depression, delineating a specific genotype–phenotype relationship in this heterogeneous disorder.

## Introduction

Apart from the evident involvement of monoamine neurotransmitter disturbances in depression, pro-inflammatory cytokine levels were repeatedly reported to be increased in depressed individuals compared to control subjects with meta-analyses also supporting this observation (Dowlati et al. 2010; Howren et al. 2009). In addition, externally administered cytokines are also able to cause depressive symptoms in animal models (Dunn et al. 2005), and depression was consistently reported as a side effect of interferon (IFN) treatment in human subjects (Bull et al. 2009; Kovacs et al. 2015). The risk of developing depression during IFN treatment was found to be dependent on a functional polymorphism in the interleukin-6 (IL-6) gene, rs1800795 (Udina et al. 2013). This polymorphism alters transcription rates of the *IL*-*6* gene in HeLA cells especially after stimulation with interleukin-1 (IL-1) or lipopolysaccharide (LPS) (Fishman et al. 1998). However, the more recent reports were inconsistent about which allele of the polymorphism causes higher expression rates (Fife et al. 2005; Kelberman et al. 2004; Terry et al. 2000). In spite of these controversies, the involvement of the rs1800795 polymorphism in the development of various disorders such as Alzheimer’s disease (Faltraco et al. 2003), schizophrenia (Zakharyan et al. 2012), and cerebral palsy (Wu et al. 2011) was also supported.

Regarding the pathophysiology of depression, it has been proposed that environmental factors—mainly stress—can exert their depressogenic effects through neuroinflammatory signalling mechanisms (McEwen 2000), and stress-induced depressive conditions were associated with significantly higher IL-6 levels (Bob et al. 2010). IL-6 was also elevated in subjects exposed to various depression-related stressful circumstances like childhood abuse, low socioeconomic status, negative social interactions, or parental loss (Slavich and Irwin 2014).

Apart from the relatively new findings about IL-6, stress and depression, IL-6 is a confirmed modulator of pain processing (De Jongh et al. 2003). In animal models IL-6 mediated neuropathic pain development (Arruda et al. 1998), C-fiber sensitization to mechanical stimuli in joint pain (Brenn et al. 2007), and enhanced migraine-like allodynia (Yan et al. 2012). Moreover, in human subjects IL-6 receptor inhibition with monoclonal antibodies showed greater therapeutic value against painful symptoms of rheumatoid arthritis compared to placebo (Smolen et al. 2008). Furthermore, IL-6 influenced the development of depression in fibromyalgia patients (Wallace et al. 2001), and a meta-analysis also demonstrated elevated IL-6 levels associated with the prevalence of depression, and inversely correlated with survival rates in patients with malignant tumors (Illman et al. 2005). The co-occurrence of these usually painful medical conditions and depression has been proposed to have a common neuroinflammatory aetiological background with the involvement of IL-6 and other cytokines (Walker et al. 2014).

Differences in the emergence of depressive symptoms during cytokine therapy was observed suggesting a primer involvement of cytokines in “somatic symptoms” (e.g., fatigue, loss of appetite, and increased pain) rather than “emotional symptoms” of depression (Loftis et al. 2013). This secondary effect on emotional aspects were proposed to be the result of tryptophan depletion caused by the primary loss of appetite, and the activation of indolamine-2,3-dioxygenase (IDO) which is the first enzyme of the kynurenine pathway, degrading tryptophan to kynurenic acid, and decreasing tryptophan levels available for serotonin synthesis (Capuron et al. 2002). Therefore, it seems to be logical to expect a slightly different symptom profile during cytokine-mediated depression.

In our study, we hypothesized that rs1800795 can interact with both physical and psychological stress factors influencing the somatic and affective depressive symptoms differently. Therefore, we tested the effect of rs1800795 polymorphisms on depressive symptom scores by two different measures, namely by the Brief Symptom Inventory (BSI), and by the Zung Self-Rating Depression Scale (ZSDS), from which the latter one has more emphasis on somatic symptoms (Gonda et al. 2005). We also examined the polymorphism’s interaction with Recent Negative Life events (RLE) and Pain Background (PBGR) information reported by the subjects.

## Methods

### Population

Collection of phenotypic and genetic data was carried out during the New Molecules in Mood Disorders (NewMood) project (Sixth Framework Program of the European Union LHSM-CT-2004-503474). 1093 volunteers provided genetic samples and phenotypic information through completing questionnaire packs delivered by postal service or personally in Budapest, Hungary. The volunteers were recruited through advertisement and general practices in universities and community-based populations (Lazary et al. 2008). Signing the official consent form was mandatory before participants entered the study. Our study was designed and performed in accordance with the declaration of Helsinki, and was approved by the local ethic committees. The statistical analysis of the population sample required exclusion of blood relatives obviously not meeting the criteria of independence from a genetic perspective. We also excluded non-white ethnic origin individuals who were represented only by eight people (1 Asian-Indian, 5 mixed, and 2 “other”), to avoid stratification bias.

### Phenotypes

We used two different outcome variables reflecting current depression in the analysis: the continuous weighted dimension scores of the depression and additional items subscales of the Brief Symptom (Derogatis and Melisaratos 1983) and of the Zung Self-Rating Depression Scale (ZSDS) (Zung et al. 1965).

Recent Life Events (RLE) were assessed by The List of Life Threatening Experiences questionnaire (Brugha et al. 1985) but only included the events that happened in the previous year. In the analysis, we used the sum of reported stressful life events in the past year. The Pain Background (PBGR) variable was derived from our background questionnaire, including the items which describe painful conditions such as migraine, back pain, or rheumatologic disorders. Pain Background was considered as a categorical variable in our analyses, with subjects reporting no painful conditions falling into category 1 and subjects reporting one or more painful condition falling into category 2.

### Genotypes

For genotype analysis, participants collected their own buccal mucosa cells with cytology brushes provided to them. DNA was extracted by a validated method (Freeman et al. 2003). The polymorphism rs1800795 of the IL-6 gene was genotyped with the Sequenom^®^ MassARRAY technology (Sequenom^®^, San Diego).

### Statistical analysis

PLINK 1.0.7 (http://pngu.mgh.harvard.edu/purcell/plink) and IBM SPSS 20.0 for Windows were used for all statistical analyses performed including linear regression, Hardy–Weinberg equilibrium determination, and Pearson correlation analysis. In the linear regression analyses age and gender of the subjects were always used as covariates. Nominal significance level was set at *p* = 0.05. Bonferroni correction for multiple testing was used to avoid bias, which reduced significance level to *p* = 2.78 × 10^−3^. Power calculations with Quanto program (http://biostats.usc.edu/Quanto.html) using the mean and standard deviation values from Table 1, and assuming the main effect of the polymorphism to explain *R*^2^ = 1 % of the variance in both ZSDS and BSI depression score, indicated a 83.6 % power to detect these effects. With the same settings but assuming RLE-genetic polymorphism interaction indicated 84.3 % power to detect effects on our outcome variables.

**Table 1:** The phenotypic variables of the population sample and their descriptive statistics

	Total *N*	Male	Female
Gender	1053	320	733
Painful conditions	140	45	95

	Total *N*	Minimum	Maximum	Mean	Standard deviation
Age	983	18	60	31.21	10.54
BSI Depression score	1049	0	4	0.554	0.683
Zung Depression score	1044	1.2	10	1.94	0.38
Recent Life Events score	1048	0	8	1.083	1.17

BSI depression score: continuous weighted dimension score of Brief Symptom Inventory’s items for depression

Zung depression score: continuous weighted dimension score of Zung Self-Rating Depression Scale

Recent Life Events: Sum of items of List of Life Threatening Experiences happening in the previous year

## Results

The examined population sample contains only European white subjects from independent families (siblings and immediate blood relatives were excluded). Out of 1053 participants, 998 provided good quality DNA and were genotyped with a call rate of 93.3 %. Due to missing phenotypic data, our analysis was carried out on 862 subjects in the case of BSI depression score, and 859 subjects in the case of ZSDS score. In our population sample, the number of female participants exceeded more than twofold the male participants’ number. The mean age of the population was 31.2 years (Table 1). There was a significant correlation between our two outcome variables the ZSDS and BSI depression scores (Pearson correlation *R* = 0.637, *p* < 0.001). The frequency of the minor (C) allele in our sample was 0.43. In contrast, the frequency of the C allele according to HAPMAP in central European population was 0.535. The rs1800795 SNP was in Hardy–Weinberg equilibrium in the sample (*p* = 1.0).

**Fig. 1:**
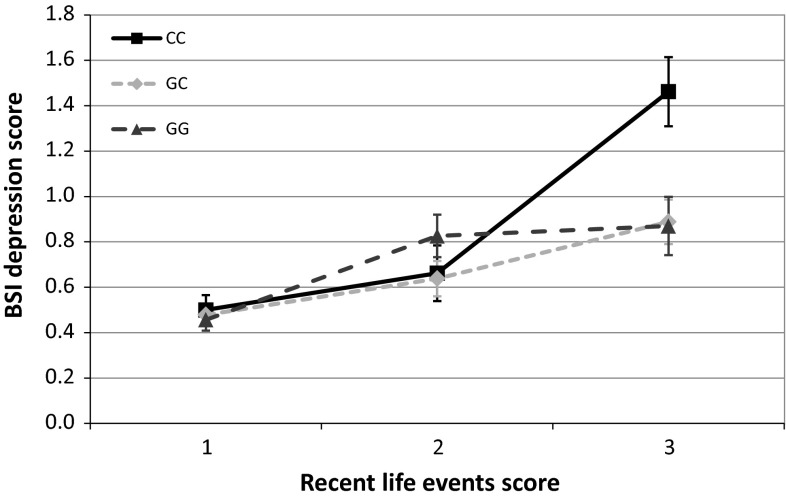
Interaction between rs1800795 and recent life events influences Brief symptom inventory depression scores. Subjects with the most Recent Life Stress (RLE) exposure and homozygote minor allele showed significantly more depression symptoms measured by Brief Symptom Inventory (BSI) compared to other groups. Significant interactions were found using additive (*p* = 0.015) and recessive (*p* = 1.19 × 10^−3^) models in linear regression analyses with PLINK 1.0.7 program

**Fig. 2:**
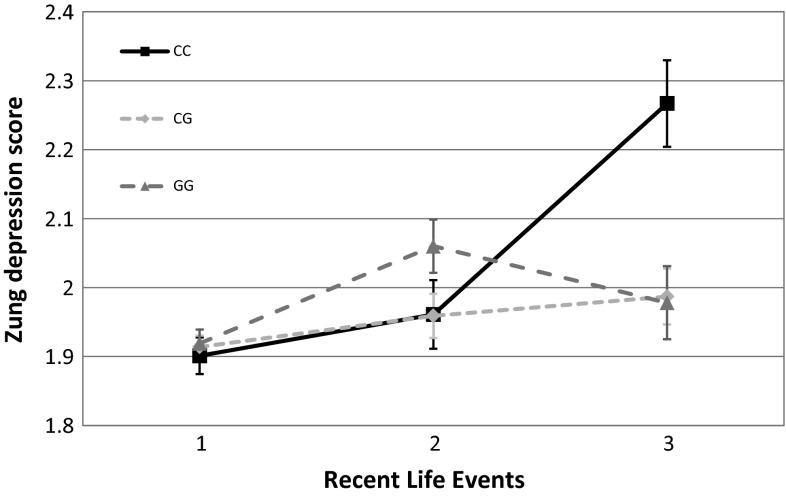
Interaction between rs1800795 and recent life events influences Zung Self-rating Depression Scale scores. Severity of life stress in the previous year (RLE) influenced rs1800795 polymorphism’s effect on depression symptoms measured by Zung Self-rating Depression Scale (ZSDS). CC genotype carriers achieved significantly higher scores in the most exposed subgroup; however, GG carriers scored slightly higher when in the mildly exposed subgroup. There is no evident difference in the least exposed subgroup. Significant interactions were found using additive (*p* = 1.17 × 10^−3^), and recessive (*p* = 2.86 × 10^−5^) models in linear regression analyses with PLINK 1.0.7 program

**Fig. 3:**
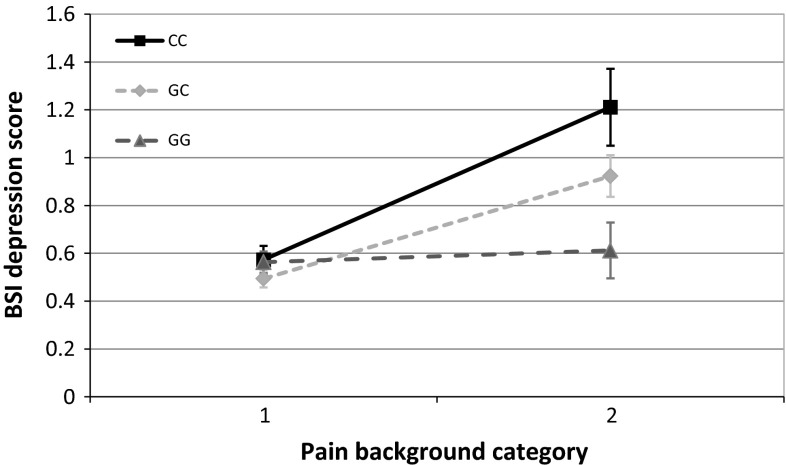
Interaction between rs1800795 and painful conditions influences Brief Symptom Inventory depression scores. Rs1800795 polymorphism in interaction with painful conditions elevates depression symptom levels measured by the Brief Symptom Inventory (BSI) in minor (C) allele carriers. Subjects in category one did not report any painful disorders, while subject group marked with two reported migraine, rheumatoid arthritis, back pain or other painful disorders. Significant interactions were found using additive (*p* = 2.96 × 10^−3^), and dominant (*p* = 4.78 × 10^−3^) models in linear regression analyses with PLINK 1.0.7 program

**Fig. 4:**
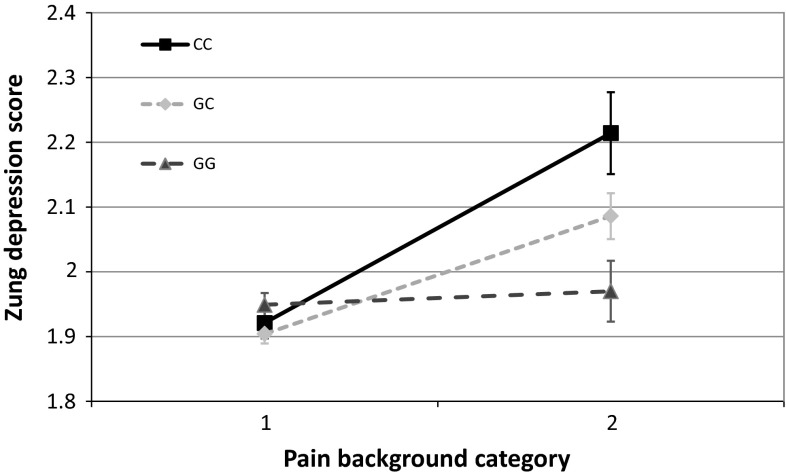
Interaction between rs1800795 and painful conditions influences Zung Self-rating Depression Scale scores. Subjects in category marked one did not report any painful conditions. Pain background category two represents exposure to painful medical conditions such as rheumatoid arthritis, migraine, or low-back pain. These conditions elevated Zung Self-rating Depression Scale Scores in minor (C) allele carriers of the polymorphism. Significant interactions were found using additive (*p* = 7.42 × 10^−4^), dominant (*p* = 2.12 × 10^−3^), and recessive (*p* = 0.021) models in linear regression analyses with PLINK 1.0.7 program

At nominal significance level, no main effect of the rs1800795 polymorphism was detected on either of our outcome variables. On the other hand, rs1800795 in interaction with RLE and assuming an additive or recessive heritability model showed significant associations both with ZSDS and BSI depression scores (Figs. 1, 2.) The interaction of rs1800795 with PBGR was also significant assuming additive and dominant heritability in the case of BSI depression score, and assuming all three heritability models (additive, dominant, recessive) in the case of ZSDS score (Figs. 3, 4). After correcting for multiple testing using Bonferroni method, the BSI depression score was only affected significantly by the rs1800795 × RLE interaction using a recessive model, while in the case of ZSDS score both additive and recessive models of rs1800795 × RLE interaction remained significant, and the rs1800795 × PBGR interaction assuming additive and dominant models also survived the correction (Table 2).

**Table 2:** Effect of the rs1800795 polymorphism and its interaction with recent life events and painful conditions on depression scores measured by the BSI and ZSDS

	ADD			DOM			REC		
	*p* value	*β*	SD	*p* value	*β*	SD	*p* value	*β*	SD
BSI Depression score									
Main effect	0.315	0.035	0.034	0.746	0.017	0.052	0.156	0.088	0.062
RLE interaction	**0.015**	0.027	0.013	0.348	0.039	0.041	**1.19** **×** **10** ^**−3**^	0.158	0.049
PBGR interaction	**2.96** **×** **10** ^**−3**^	0.307	0.103	**4.78** **×** **10** ^**−3**^	0.422	0.149	0.06413	0.346	0.186
ZSDS score									
Main effect	0.971	0.0005	0.014	0.571	−0.01	0.021	0.459	0.018	0.025
RLE interaction	**1.17** **×** **10** ^**−3**^	0.036	0.011	0.167	0.023	0.017	**2.86** **×** **10** ^**−5**^	0.085	0.02
PBGR interaction	**7.42** **×** **10** ^**−4**^	0.139	0.041	**2.12** **×** **10** ^**−3**^	0.186	0.06	**0.021**	0.171	0.074

Significant interactions are highlighted in bold (*p* < 0.05)

BSI depression score was measured by the Brief Symptom Inventory depression subscale. ZSDS score was measured by Zung Self-Rating Depression Scale. RLE: Recent life events measured by List of Life Threatening experiences, PBGR: Painful conditions measured by our background questionnaire. *SD* standard deviation, *ADD, DOM, REC* additive, dominant, recessive heritability models

## Discussion

Based on our results, the rs1800795 polymorphism, situated within the promoter region of the *IL*-*6* gene significantly interacted with both psychological and physical stress factors on depressive symptoms. In addition, this interaction effect was stronger on depressive symptoms measured by the Zung Self-Rating Depression Scale compared those measured by the Brief Symptom Inventory suggesting that IL-6 plays a more important role in the development of somatic depressive symptoms than emotional-cognitive symptoms.

### Somatic and psychological stress factor interactions

The differences between the intensity of the RLE and PBGR interactions in our sample were not relevant, suggesting that both of them are important in the development of depressive symptoms. However, it is interesting that RLE interactions preferred additive and recessive models, while PBGR interactions preferred additive and dominant models. Thus, painful conditions might be able to induce depression in CG heterozygotes, where the expression rate just slightly changed, while only homozygous CC carrier status causes vulnerability to other life stressors, where the *IL*-*6* expression rates changes more considerably. Based on the previous studies which investigated plasma IL-6 levels in depression and during stressful experiences we can assume that our risk variant, namely the C allele, might induce over expression of *IL*-*6* and thus increase depressive symptom scores (Howren et al. 2009).

Initially the major allele (G) of rs1800795 was found to be the higher IL-6 expressing variant (Fishman et al. 1998). This SNP is located at the promoter region of the IL-6 gene and changes its transcription rate by modulating the transcriptional factor binding capability of the promoter sequence (http://snpinfo.niehs.nih.gov/cgi-bin/snpinfo/snpfunc.cgi). Recently it has been also suggested that this polymorphism is part of a functional haplotype, exerting its effect in a non-independent manner on gene expression (Fife et al. 2005). Although unfortunately we only measured rs1800795 and no other haplotype tagging SNPs in this region these recent findings might explain why we found the minor C allele to be the risk variant on depression in interaction with both of our stress factors.

### Differences in the sensitivity of outcome phenotype measures

Generally, we found ZSDS more suitable for the detection of the *IL*-*6* polymorphism’s effect on mood disturbances. The differences in significant hit numbers were minimal on nominal significance level; however, after correction for multiple testing with Bonferroni method, only one significant hit remained in the BSI depression group, while five of them survived the correction in the ZSDS group. The differences indicate that somatic symptoms of depression, captured more expansively by ZSDS compared to BSI scale are more responsive to the interactions of rs1800795 polymorphism. ZSDS has a well-established somatic subscale (Gonda et al. 2005; Kitamura et al. 2004) and was also more sensitive to assess depression risk in spinal cord injury patients compared to BSI depression scale (Tate et al. 1993). Moreover, changes in cytokine expression profile with increased ZSDS score were demonstrated in depressed patients with significant differences in childhood trauma-exposed versus non-exposed groups of patients, suggesting that symptoms measured by the ZSDS reflect psychological stress-induced cytokine-mediated pathological processes (Lu et al. 2013). In summary, our results concerning the rs1800795 polymorphism strengthen earlier findings which support that ZSDS is a superior tool for measuring cytokine-mediated depression when psychological or physical interacting factors were present.

### Psychobiological implications of the results

Since IL-6 has a prominent role in cognitive function (McAfoose and Baune 2009), and in the internalization of various stress types (Slavich and Irwin 2014), it is possible that IL-6 is involved in depression-specific changes of the brain structure. Most studies reported a neurodegenerative effect of IL-6 (Marsland et al. 2008; Vallieres et al. 2002), however, the earlier findings assigned neuroprotective features to IL-6 (Tilg et al. 1997; Wagner 1996). The neuroprotective function is supported by an MRI study which reported increased hippocampal gray matter volumes in carriers of the G allele of rs1800795 polymorphism (Baune et al. 2012). As mentioned above there is a well-established hypothesis that environmental stress factors can affect depressive states by neuroinflammatory mechanisms such as hypothalamus–pituitary–adrenal (HPA) axis hyperactivity (Mokrani et al. 1997), or activation of the kynurenine pathway (Miura et al. 2008). However, based on the available literature and our results, IL-6 seems to have a two-faced effect on depression phenotype. Even though it was found to be elevated in the peripheral blood serum samples of depressed patients by a meta-analysis (Dowlati et al. 2010), it is necessary for healthy brain development and for synaptic plasticity (Molina-Holgado and Molina-Holgado 2010). Considering these findings and also that rs1800795 is probably exerting its effect on IL-6 expression rate through a functional haplotype rather than alone (Fife et al. 2005), it seems unlikely to find either allele of the rs1800795 polymorphism to be exclusively associated with a depression phenotype.

### Limitations

In the analysis we could only examine the effect of the rs1800795 polymorphism; however, it has been proposed to be part of a functional haplotype (Fife et al. 2005). The gender distribution of our population sample was not balanced and also the sample size is considered relatively small nowadays. Our analysis considered the interaction effect of rs1800795 with painful conditions and recent negative life events, although it is possible that many other factors exist which can modulate the effect of rs1800795, such as social support, childhood adversity, or socioeconomic variables. Also some of the findings did not meet the criterion of Bonferroni corrected significance threshold; so further studies are required to confirm the validity of them. We measured the outcome and interacting variables by self-report questionnaires which can produce biased results.

## Conclusions

Rs1800795 polymorphism in interaction with both physical and psychological stress factors influenced depressive symptom phenotypes measured by the Zung Self-Rating Depression Scale and the Brief Symptom Inventory, but no main effect of the polymorphism was detectable. This suggests that further investigations should also consider including interacting factors, whether they are painful disorders or negative life events, when they examine the effect of rs1800795 on depression. Our study also supports the hypothesis that cytokines affect physical and somatic aspects of depression to a greater degree than the emotional aspects, therefore using the proper outcome measure such as the ZSDS might significantly improve the success rates of further studies investigating the effect of cytokines on depression.
